# Pharmacogenomic technologies: a necessary "luxury" for better global public health?

**DOI:** 10.1186/1744-8603-7-30

**Published:** 2011-08-24

**Authors:** Catherine Olivier, Bryn Williams-Jones

**Affiliations:** 1 Bioethics Programs, Department of Social and Preventive Medicine, Université de Montréal, Montréal, Canada

**Keywords:** Pharmacogenomic technologies, drug development, health innovations, global, public health, developing world populations, University of Toronto Joint Centre for Bioethics criteria, inequity, luxury

## Abstract

**Background:**

Pharmacogenomic technologies aim to redirect drug development to increase safety and efficacy of individual care. There is much hope that their implementation in the drug development process will help respond to population health needs, particularly in developing countries. However, there is also fear that novel pharmacogenomic drugs will remain too costly, be designed for the needs of the wealthy nations, and so constitute an unnecessary "luxury" for most populations. In this paper, we analyse the promise that pharmacogenomic technologies hold for improving global public health and identify strategies and challenges associated with their implementation.

**Discussion:**

This paper evaluates the capacity of pharmacogenomic technologies to meet six criteria described by the University of Toronto Joint Centre for Bioethics group: 1) impact of the technology, 2) technology appropriateness, 3) capacity to address local burdens, 4) feasibility to be implemented in reasonable time, 5) capacity to reduce the knowledge gap, and 6) capacity for indirect benefits. We argue that the implementation of pharmacogenomic technologies in the drug development process can positively impact population health. However, this positive impact depends on *how *and for *which purposes *the technologies are used. We discuss the potential of these technologies to stimulate drug discovery in the case of rare (orphan diseases) or neglected diseases, but also to reduce acute adverse drug reactions in infectious disease treatment and prevention, which promises to improve global public health.

**Conclusions:**

The implementation of pharmacogenomic technologies may lead to the development of drugs that appear to be a "luxury" for populations in need of numerous interventions that are known to have a demonstrable impact on population health (e.g., secure access to potable water, reduction of social inequities, health education). However, our analysis shows that pharmacogenomic technologies do have the potential to redirect drug development and distribution so as to improve the health of vulnerable populations. Strategies should thus be developed to better direct their implementation towards meeting the needs and responding to the realities of populations of the developing world (i.e., social, cultural and political acceptability, and local health burdens), making pharmacogenomic technologies a necessary "luxury" for global public health.

## Background

Luxuries are generally defined as goods that respond to *wants *rather than *needs*, and are more often associated with things that are *frivolous *rather than those that constitute a *necessity*. An economic concept, a "luxury good" refers to goods for which the costs, in terms of household expenditures, rise more rapidly than income [1]. Consequently, sports cars, expensive jewellery, spacious homes and rare foods or wines are all goods that we voluntarily classify as luxuries in richer countries. However, defining goods as luxuries may depend in important ways on the cultural and social realities in which the goods are distributed. Access to certain types of housing, food or education even at their simplest level may, for example, constitute luxuries in the context of low-income countries. The distribution of certain goods in a context of significant global inequalities can raise doubts regarding their uselessness, and thereby change the perception of luxury or necessity in a given context.

Healthcare services and goods are usually perceived of as *needs*, and so health innovations such as genomic technologies, which apply information obtained from the genome (complete genetic material) of individuals or organisms to medical innovations, often benefit from wide public support. However, international health inequities raise questions about the global acceptability of many health innovations. Inequities in health are described as inequalities that can be considered both unjust and avoidable [2]; and they are increasingly affecting the least favoured populations of the world. Although discussions are still ongoing in the bioethics literature with regards to *how *and *why *inequalities can be declared unjust or avoidable, these inequities can often be attributed directly to various economic, political and social determinants. It is argued that the reduction of health inequities may more easily result from increased access to basic needs (e.g., food, potable water, shelter), than from public investments in health innovations that show low potential for responding to pressing population health needs [3,4]. Thus, although a good deal of hype surrounds the potential of emerging genomic technologies to improve public health, these might be reasonably considered a luxury for populations of the developing world that have clearly identified and pressing needs for basic public health interventions.

Genomics medicine as a field of study emerged with the sequencing of the human genome. It is considered by many to have the potential not only to revolutionize the way we do medicine, but also to lead to technologies that will help reduce the significant gap in life expectancies that exist amongst various populations of the world [5]. Pharmacogenomics, which integrates genomics information in the drug development and prescription processes, is one example of such technologies. The potential of genomic technologies to revolutionize medicine derive from two important observations: 1) the implementation of prior technological innovations in medical practice have been a major contributor to the decrease in global mortality and increase in life expectancy for the period spanning 1960 to 1990 [6], and 2) genomics knowledge can be understood as a global public good that can have a potential positive impact at the international level.

Global public goods are goods that are both non-excludable (can be enjoyed by all, e.g., water) and non-rivalrous (can be consumed by many without suffering from depletion, e.g., air), and made public across national borders [7]. One such public good is scientific knowledge, and for our purposes, genomics. As genomics research and medicine result from population-based knowledge and applications, and because the field has from the outset transcended national or institutional borders (e.g., international collaborations, publishing in scientific journals, submitting raw data to public Internet databases), the "publicness" of genomics has also shifted from a national to a global level [7]. Genomics knowledge and resulting technologies should thus be seen as a public good to benefit all of humanity - and especially those populations most in *need *- and not simply as a luxury resource to meet the *wants *of populations in developed countries.

### Assessing the Potential of Genomics Technologies in Public Health

In 2002, the University of Toronto Joint Centre for Bioethics published a study that aimed to identify the Top 10 biotechnologies most likely to improve health in developing countries in the next 5 to 10 years [8]. Their study was the result of an initiative that followed the 2000 United Nations presentation of the Millennium Development Goals (MDG), which aim to significantly reduce poverty and improve health at the global level by 2015. The Toronto group hoped to identify technologies associated with genomics that could contribute to global efforts to reach the MDG. Their study provides clear indications on the types of technologies that should benefit from public investment in order to reduce global inequities in health. These include [8]:

1. modified molecular technologies for affordable, simple diagnosis of infectious diseases,

2. recombinant technologies to develop vaccines against infectious diseases,

3. technologies for more efficient drug and vaccine delivery systems,

4. technologies for environmental improvement (sanitation, clean water, bioremediation),

5. sequencing pathogen genomes to understand their biology and to identify new antimicrobial drugs,

6. female-controlled protection against sexually transmitted diseases, both with and without contraceptive effect,

7. bioinformatics to identify drug targets and to examine pathogen-host interactions,

8. genetically modified crops with increased nutrients to counter specific deficiencies,

9. recombinant technology to make therapeutic products more affordable,

10. combinatorial chemistry for drug discovery.

The Toronto study was initiated in 2001, and the 5 to 10 years period during which the technologies identified should have an impact on population health has now passed. Given technological developments in the last decade, a similar study today would probably lead to the identification of different technologies, and include, we suggest, pharmacogenomic technologies. Pharmacogenomic technologies, which integrate genomic information obtained following the completion of the human genome project and other genomics initiatives (e.g., the HapMap project) in drug development and distribution protocols, hold the promise of reducing adverse drug effects or reactions (ADRs) that result from genomic polymorphisms that affect individual drug metabolism and elimination [9]. These technologies include pharmacogenomic tests for predicting individual drug response and drug testing for their genomic efficacy, leading to the development of pharmacogenomic drugs. Their implementation in the healthcare context promises to increase safety and efficacy by personalising the treatment of diseases and health related problems [10,11], and much hope - and hype - has accompanied their development [12].

At the global level, it has been suggested that pharmacogenomic technologies hold the potential to improve knowledge about neglected and orphan diseases, and to lead to the development of novel drugs that could improve global population health [13-15]. Not surprisingly, initiatives have emerged in some low or middle-income countries (e.g., Thailand, Mexico and India) to integrate pharmacogenomics into their efforts to meet the MDG [16-18]. The potential of pharmacogenomic technologies to positively impact global public health depends on their development and application at both national and international levels, something that necessitates national and international policy-making, health related agreements and public investment. A systematic evaluation of the potential of these technologies is thus needed to help identify issues and considerations susceptible to impact their effective implementation. Such an evaluation can help determine whether pharmacogenomic technologies should be seen as a "luxury" or a "necessity" for populations of low- and middle-income countries (can they really improve global public health?), and thus empower local and international decision makers to make informed choices about the funding (or not) of pharmacogenomic innovations.

This article is the first step in a study that seeks to evaluate the potential that pharmacogenomic technologies hold to address the needs of population health and reduce global injustice in health through access to safe and effective medicines. The criteria chosen for the current evaluation are the ones initially described by the Toronto group and the United Nations report on Genomics and Global Health in 2004. They can be summarised in six major points [5,8]:

1. impact of the technology (capacity for improvement),

2. technology appropriateness (affordability, adjustability to health care settings, as well as social, cultural and political acceptability),

3. capacity to address local burdens,

4. feasibility to be implemented in a reasonable time,

5. capacity to reduce the knowledge gap (provide knowledge advancement), and

6. capacity for indirect benefits (e.g., environmental or social).

Each of these criteria will be examined in turn and applied to pharmacogenomic technologies. This analysis does not aim to *prove *that current pharmacogenomic technologies *will *or *can actually *meet population health needs. Instead, the aim is to provide a structured analysis of the *promises *of pharmacogenomic technologies in order to clarify what they *would have to accomplish*, and the associated challenges, in order to contribute to improving population health and global justice.

## Discussion

The current international public health context is one where many populations have significant problems in accessing appropriate healthcare services and medications, thus constituting a major global injustice. This injustice is further exacerbated by the fact that technological and medical innovations have historically been developed for populations of high-income countries in the developed world, thereby contributing to disparities in world health. This is particularly apparent in the "90/10 gap", where 90% of investment in drug development is directed towards meeting the needs (or wants) of developed world populations, while only 10% is directed towards the needs of developing world populations [19,20]. In 2011, the CIA World Factbook estimates a 40 year difference in the life-expectancies between the world's richest and poorest populations; the countries with the highest life-expectancy are Andorra and Japan at 82 years, while the lowest life-expectancy can be found in Angola, at 38 years [21]. Ten countries are listed as having a life-expectancy of 50 years or less, 9 of which are from the African continent [21].

This population life-expectancy gap clearly demonstrates that certain populations, but not others, have the tools and opportunities that enable them to lead or expect to lead longer and healthier lives. Moreover, these disparities inequitably and unjustly affect the peoples of the developing world. The root causes are numerous and tied to national realities that make it exceedingly difficult to address at a global level [20]. Nonetheless, there are some tools or opportunities developed and provided by groups or institutions with global activities that can have an impact on local or national public health realities. One such actor is the pharmaceutical industry, which has benefited from the globalization of markets and is constantly increasing drug distribution around the globe [22]. Among the tools developed in this industry are pharmacogenomic technologies and drugs. But, it is not clear yet how pharmacogenomic technologies can or will be applied in today's global health context. *In brief, can the implementation of pharmacogenomic technologies in the drug development process contribute to meeting the health needs of vulnerable populations of the developing world? *In working through the Toronto group's framework, the present analysis evaluates the potential of pharmacogenomic technologies and identifies means by which various actors in pharmacogenomics can begin addressing pressing global public health needs.

### 1) Potential impact of pharmacogenomics

The idea that genetics might have an influence on individual responses to various medications is far from new. It was Archibald Garrold, at the beginning of the 20^th ^century, who first described chemical individuality in drug response [23]. Since then, numerous examples of varying individual responses to specific drugs have been identified. A now classic case is Merck Frosst's star product Vioxx, withdrawn from the market in September 2004 following evidence of significant ADRs. This non-steroidal anti-inflammatory drug, introduced in the late 1990s as a replacement for classic anti-inflammatory drugs such as ibuprofen, is part of the Cox-2 inhibitor family which has since been shown to have variable effects on the cardiovascular system and blood pressure, notably increasing the risk of heart attacks and strokes in some individuals [24,25].

Diversity in drug reaction due to genetic differences is a major factor in ADRs, in combination with physiological (weight, gender and age), environmental (pollution and diet) and social (wealth, education and family) factors that are susceptible to impair treatment success and that can even lead to death [26]. The relative efficiency of a specific drug or treatment commonly used in hospitals has been shown to vary between 30 and 70% [27]. A milestone publication on the topic demonstrated that in the United States alone, there are approximately 2 million hospitalisations and 100,000 deaths per year attributable to ADRs [28]. A more recent meta-analysis suggests that ADRs account for approximately 5% of hospital admissions in Western countries [29], with higher rates among the elderly (up to 16.6%) [30]. Interestingly, a 2005 study of emergency department admissions in India found that between 6 and 7% of admissions were due to ADRs, and that in most cases (60%) these were avoidable [31]. These studies show that the occurrence of ADRs constitutes a significant population health problem worldwide.

In the US, close to 50% of the population uses at least one prescription drug on a regular basis; the most common drugs are for treating asthma in children, and antidepressants and cholesterol lowering drugs [32]. In the developing world, prescription drug sales have been on the rise and IMS Health (Intercontinental Medical Statistics) estimates that the $67 billion in sales in 2003 will jump to a staggering $265 billion by 2013 [33]. Pharmaceutical companies such as Pfizer or Novartis are building on the promise of market expansion and have a growing number of sales representatives marketing their star products, such as Lipitor, to doctors and clinics in low-income countries [33]. Considering the importance that prescription drugs play in modern medical and public health practices, tools to enable more effective evaluations of the safety and efficacy of a drug in a particular population, or for specific individuals, in order to reduce the occurrence of ADRs could have an important positive impact on global public health.

In taking into account the genetic factors susceptible to influence the metabolic outcome of specific drugs for individuals or for segments of the population, pharmacogenomics promises to contribute to better success in disease treatment and an increase in the general health of the population [34]. For example, the integration of genomic information relating to drug metabolism during the drug development process will hopefully enable the pharmaceutical industry to develop drugs better suited for the treatment of diseases in particular ethnic groups or for a portion of the population sharing similar genomic characteristics. Moreover, a shift towards the development of pharmacogenomic drugs may justify focusing research and development efforts on diseases or biological susceptibilities (ADRs, response to pathogens or predispositions to diseases) that are characteristic of specific populations. This can be extremely important for populations disfavoured by the current 90/10 context in drug development, namely the populations of low and middle-income countries.

The implementation of pharmacogenomic technologies in the drug development and distribution context could thus have a positive impact on population health. But these technologies may also raise serious ethical concerns in other spheres of society (e.g., social, cultural or political) that could undermine the appropriateness of these technologies.

### 2) Appropriateness of pharmacogenomic technologies

The second criteria used by the University of Toronto Joint Centre for Bioethics study for the evaluation of novel technologies most susceptible to impact health in developing world countries is the technology's *appropriateness *[8]. Daar and colleagues describe this criterion in relation to the technology's affordability, robustness, adjustability to the local contexts and social, cultural or political acceptability.

#### Affordability

Affordability is clearly a major concern in a developing world context. But it is not sufficient to say that "the drugs are too expensive"; drug costs, and thus their affordability, are associated both with the social cost of investing in these technologies (upstream innovation) and the resulting (downstream) cost of then integrating these technologies in the drug development and distribution process. One third of the world's population currently does not have access to basic essential medicines [35], and this proportion extends to half the population in the poorest regions of the globe (e.g., certain regions in Africa and Asia). Not surprisingly, then, drug accessibility constitutes an important element in preoccupations about global justice [20]. Drug inaccessibility is in large part due to the fact that many drugs are simply too costly for people to purchase, especially those living in low- or middle-income countries that invariably lack universal healthcare insurance programs.

This situation is exacerbated by the defence - on the part of the pharmaceutical industry and governments of developed countries - of strong intellectual property rights (IPRs), i.e., patents. IPRs are presented as an essential constituent in the drug development process, a means for companies to protect (and recoup) their economic investment in an innovation (e.g., a drug), thereby making it worthwhile for pharmaceutical companies to invest in research and development of new drugs. The broad and international application of strong IPRs for medicines followed the 1995 Trade Related Aspects of Intellectual Property Rights agreement (TRIPS) of the World Trade Organization (WTO) [36]. These strong IPRs combined with pricing practices designed for the wealthy developed countries can have a significant negative impact on drug distribution, making novel and effective drugs simply unaffordable and thus inaccessible for people in the world's poorest countries.

Similarly, the ways that pharmaceutical drugs are regulated in different national contexts can have important repercussions on the accessibility and affordability of novel drugs for populations in need [37,38]. The lack of regulations on the pricing or reimbursement of drugs made expensive by the implementation of novel pharmacogenomic technologies in their development process can limit the potential use of these new drugs in low and middle-income countries. The development of new technologies, such as pharmacogenomics, often requires sophisticated equipment, infrastructure and specialised human resources. Unfortunately, high tech equipment and knowledgeable human resources are two elements that can be very costly for research groups and companies interested in investing in pharmacogenomic technologies. This situation increases the incentives for pharmaceutical companies to raise the costs of drugs produced using such novel technologies in order to offset their initial investment. When combined, these costs constitute a significant barrier to the provision of pharmacogenomic drugs in the developing world.

The lack of comprehensive and universal public health insurance in most developing world countries means that these populations bear the financial burden of ill health. Out-of-pocket health expenditures are situated at around 50% in low-income countries [39], among which the greatest part is directed towards drug purchases (e.g., 70% in India [40] and over 80% in certain Sub-Saharan countries). These purchases constitute the second most important household expenditure, after food [41,42]. For example, the mean direct costs of the three major diseases affecting populations of the developing world (malaria, tuberculosis and HIV/AIDS) represent between 2.5 and 7% of household income [43]. Furthermore, self-medication and over-the-counter drug purchases are extremely frequent, increasing the pharmaceutical drug share of household expenditures [44]. Thus, even though funding of health sectors has increased 88% between 1995 and 2006 in most developing nations (excluding Sub-Saharan Africa) [45], the financial burdens of meeting individual health needs inevitably reduces health benefits for these vulnerable populations. Increasing the financial burden of drugs for individuals (who are paying out-of-pocket) or government organizations (e.g., public health programs) with limited funds could be an important disincentive for doctors and pharmacists to prescribe or sell novel drugs, because these prescriptions would not be filled nor their costs reimbursed by state health agencies. As a result, novel medicines would prove to be unaffordable and thus inaccessible for the populations of low and middle-income countries, thereby limiting the potential benefits of pharmacogenomic technologies for these populations.

#### Robustness

Robustness is a concept linked to the capacity of a system to survive or resist unpredictable perturbations. It is a difficult concept to assess at the social level, but can be evaluated through the identification of variables that may affect its vulnerability within a given setting (e.g., healthcare services) [46]. When it comes to implementing novel technologies in healthcare services, many variables can affect long term efficiency and thus impair robustness. Among these variables, funding availability and allocation are significant factors since they can dictate priorities and shape the direction of technological development, elements that impact the eventual sustainability and success of a given technology, and thus its robustness. Insufficient funding for drug research and development (R&D) can, for example, make it more difficult to develop novel drugs that can better meet population health needs.

Funds devoted to drug R&D take a variety of forms, the principal ones being: public funds, philanthropic donations, government investment or tax deductions [47]. To increase access to drugs for their populations, certain low- (e.g., Kenya) and middle-income countries (e.g. India, Thailand, Brazil and South Africa) have invested in the establishment of national pharmaceutical industries [48]. These local pharmaceutical companies invariably focus on producing cheaper generic drugs for their populations, but are also involved in developing new drugs. Indian companies, for example, account for 2% of drug patents awarded in the US and almost 2% in Europe [49].

Although pharmaceutical companies around the world have benefited from a range of funding sources, including government agencies promoting public health oriented R&D agendas, actual investment in R&D geared at meeting the needs of low- and middle-income country populations has not risen significantly [47]. In India, the proportion of funds allocated for R&D for diseases predominant in the developing world has actually dropped from 16% in 1998 to 10% in 2003 [49]. The appeal of greater sales and profit in high-income countries may thus divert drug developers' intentions to respond to the needs of poorer populations. Finally, political and economic pressures, such as the global financial crisis that weakened national economies in Europe and North America since 2008, may also contribute to a decrease in funds available from national governments and donors for drug R&D.

There are not many indications that the implementation of pharmacogenomics in the drug development process will change the current trend in R&D funding or orientation. Higher costs associated with their development, as described in the previous section, may significantly impair their long term use and thus reduce their overall robustness.

#### Adjustability to local contexts

The current drug R&D context already strongly disfavours developing world populations. A study of the number of publications found in the major scientific journals (via *Medline*) demonstrates that this situation is not changing; in 2004 only 1.25% of published clinical studies related to tropical diseases [49]. It has been proposed, however, that pharmacogenomic technologies may help reduce both the cost and time needed for drug development and maximize drug potential through reattribution of medical purpose for previously rejected drugs [50]. Thus, it is not obvious that these novel technologies will necessarily increase the market cost for novel drugs. As already mentioned, these promises have led some authors to argue that pharmacogenomic technologies can contribute to the reduction of global health inequities [15,51,52].

Pharmacogenomic technologies have the potential to help scientists identify individuals likely to respond positively to a given class of drugs. This capacity can enable researchers to better circumscribe the sample population used for clinical testing, thereby reducing sample sizes and better adjusting the study to local contexts. It also reduces the period of time during which clinical tests need to be conducted by ensuring better response possibilities in the sample population. Indeed, the possibility of screening participants for certain genomic predispositions to drug response (e.g., testing for specific major drug metabolism enzyme alleles) could provide companies better chances of obtaining a positive response in the majority of individuals during clinical trials. Such positive responses would then 1) reduce the number of trials needed to support the potential benefit of tested drugs, 2) reduce the amount of time required for these clinical trials, 3) facilitate the accreditation of new drugs by national review agencies such as the US Food and Drug Administration (FDA), and ultimately 4) help reduce the cost of drug development and thus the overall price of drugs on the market [53].

However, the conduct of clinical drug trials in developing countries raises a host of ethical and practical challenges, and pharmacogenomics research would likely be no different. Notable concerns include: the ability to obtain valid informed consent [54,55]; the exploitation of vulnerable individuals who bear the risks and burdens of research while being unlikely to benefit from the results [56,57]; the scientific validity of clinical studies conducted in contexts that lack appropriate regulation and governance mechanisms [58]; and the respect of the research participant's right to withdraw from clinical trials [59].

Finally, the integration of pharmacogenomic information in the context of clinical trials could be used to unfairly exclude groups of individuals with greater risks of developing ADRs to the drug being tested. On the one hand, severe ADRs in individuals may not be assessed in clinical trials that preselect participants based on their potential to respond positively to a given drug. Alternative drug development may be abandoned for the least interesting vulnerable groups of individuals that prove at higher risk of ADRs for the major drug being developed. Pharmaceutical companies could thus use this information to take important decisions on the withdrawal from the development pipeline of drugs that are shown to have limited market potential (the population who would benefit is too small, too poor, etc.), even though these could prove to be safer and more efficient for the vulnerable populations of low and middle-income countries.

#### Social, cultural or political acceptability

The development of drugs that are better suited to the local context in terms of population and cost can increase the possibility for populations of developing countries and their governments to purchase much needed drugs. The implementation of pharmacogenomic technologies in the drug development and distribution context can in this sense be considered socially and politically acceptable; however, it is not obvious that the implementation of such technologies will necessarily respond to norms of cultural acceptability.

The first drug labelled for a racially identified population was BiDil, approved in 2005 by the FDA for treatment of chronic heart failure in individuals of African-American origin [60]. This drug was shown to reduce mortality by 43% and hospitalisations by 39% in African-Americans, but showed no specific increase in efficiency for other racial groups [61]. At first glance, the development of a drug that is specific to a particular ethnic or racial group, and which addresses an important health need, is a perfect example of progress towards developing personalised medicines that meet the needs of specific communities, and not only the general population. However, important critiques have been raised about the BiDil study's validity (e.g., regarding participant selection procedures) [62-64], and its distribution in a race-based manner [65-67]. Notably, the high cost of BiDil raises questions about the utility of developing drugs based on race or ethnicity that may provide more personalised treatment to individuals, but prove to be inaccessible to those populations most in need [68].

The BiDil case also highlights more general concerns about ethno-typing in pharmacogenomics studies. In focusing on rough ethnic or racial categories (instead of more neutral genomic or biomarker categories), studies in pharmacogenomics may lead to undue and incorrectly linear associations of specific genetic dysfunctions with ethnic groups, thereby contributing to or augmenting pre-existing racial discrimination and ethnic stigmatization. Furthermore, instead of using pharmacogenomic information on a vulnerable population to direct drug development to meet their specific needs, such information can be used as a population exclusion criteria. That is, companies or governments might want to exclude certain populations from studies because they are vulnerable (i.e., are socially or politically sensitive) and not sufficiently wealthy to afford the resulting product.

It is also important to consider social and cultural factors related to the perception of Western medicines in comparison with traditional forms of medicine. Specifically, people may have very different views - informed by religious/spiritual, cultural or social factors - regarding the utility and function of pharmaceutical drugs [69], who should prescribe them [70], who they are appropriate for, and how they should be used [71,72]. Such factors will likely have an impact both on participation in clinical trials of pharmacogenomic technologies, and in the uptake of any resulting pharmacogenomic drugs. Further, in contexts where there is little or no universal drug insurance - the case for most low- and middle-income countries - individual consumers will often be the final decision maker (purchasing the drug from a pharmacist or street vendor) about which drug is appropriate for them and their families, regardless of the actual clinical indication of pharmacogenomic specificity.

Possible racial discrimination and stigmatization in the drug development and distribution process, along with important variation in how peoples perceive novel Western medicines, place pharmacogenomics in an ambiguous situation with regards to the ability to address inequities in global health. The benefits gained from the integration of pharmacogenomic technologies into medical practices might thus be accompanied by significant social disadvantages that actually impair the capacity of these technologies to address local health burdens. Companies and governments investing in pharmacogenomics must therefore pay careful attention to both the needs and the socio-cultural contexts of the populations that they are seeking to help.

### 3) Capacity to address local burdens

Pharmacogenomic technologies may provide powerful tools to *enable *the pharmaceutical industry to develop drugs that are specific for diseases that afflict the developing world population, promising important modifications to the current drug development and distribution system. In targeting sub-groups or populations, these technologies have the potential to fragment the market for innovative drugs (many sub-populations, many competing drugs) and so force pharmaceutical companies to move away from their traditional blockbuster (1 disease = 1 drug) model [52]. It is thus not clear that these technologies will favour a *change *in the current philosophy of drug discovery that is focused on meeting the demands of wealthy countries.

The development of a multiplicity of pharmaceutical drugs that can respond to novel disease niches could help compensate for this market segmentation, creating new market opportunities for the various actors in drug development and distribution [52]. Obviously such segmentation does not guarantee that drug development will be geared towards meeting the needs of developing country populations. However, it may make smaller or less profitable markets, such as low and middle-income countries, more attractive for drug developers [52]. This is especially true when population size is taken into consideration, since low- and middle-income countries account for most of the world population. Segments or sub-groups of the population in these contexts have the potential to be greater in number than in developed countries and so can represent more interesting market shares. Nonetheless, pricing models (drugs priced for developed world markets) would also need to change for the benefits associated with market segmentation to be actualised. In reality, introducing pharmacogenomic technologies into drug R&D processes may increase the cost (and thus market price) for novel drugs and lead some pharmaceutical companies to choose not to invest in sectors with less attractive market potential, namely, lower cost drugs addressing the needs of developing world populations. Pharmacogenomic drugs will thus become costly "luxuries", only available to wealthy members of genomic sub-populations, thereby contributing to a widening of the already significant global gap in access to safe and effective medicines.

The ongoing HIV/AIDS epidemic and access to anti-retroviral therapy (ART) is one example of the current challenges in making safe and effective medicines accessible. The highest HIV prevalence in the world is in developing countries, with 68% of HIV-infected individuals in the Sub-Saharan region [73]. ARTs are currently the most effective means of treating HIV-infected individuals, but they are very expensive and so unaffordable for most. Regional governments and non-governmental organisations (NGOs) in a number of countries (e.g., Uganda, Brazil, Thailand) have established universal drug plans for ART. Unfortunately, a great deal of stigmatization and discrimination is still associated with HIV+ status, which negatively impacts compliance with ART protocols and thus limits efforts to contain the epidemic.

It is also well documented that ARTs induce important ADRs in certain individuals, ranging from coetaneous reactions (rash) to severe gastrointestinal or liver reactions [74]. As a consequence, 25% of patients do not adhere completely to their treatment plan, increasing risks of HIV transmission in the general population [74]. These variations in individual response to ART are increasingly recognised as being associated with genetic factors [75]. As such, pharmacogenomic technologies could help public health professionals better target specific HIV treatments for infected individuals, and also facilitate the development of drugs that are better suited to sub-populations based on their shared genomic background. Pharmacogenomic technologies could then improve patient experiences and thus compliance with ART, and compliment social and cultural efforts to reduce stigmatisation of HIV+ individuals.

As suggested in the previous section, reductions in the costs of drug R&D could constitute an important incentive for companies to invest in pharmacogenomics research that targets diseases that are predominant in the developing world but costly to treat (such as HIV), as well as those that have not yet been well explored (e.g., orphan or neglected diseases). For example, it has been suggested that pharmacogenomics could be used in drug response predictions for treatments for Crohn's disease [76]. Crohn's disease is a disorder characterized by lifelong inflammation of the intestinal *muccosae *for which no cure presently exists, and is sometimes classified as a rare disease (e.g. Orphanet database on rare diseases and orphan drugs [77]). In the US, the prevalence of Crohn's disease is estimated to be approximately 500,000 individuals. This prevalence excludes it from the classical definition, which defines an "orphan disease" as a disease affecting less than 200,000 individuals. Crohn's is nonetheless not a very common disease. Regular treatment of individuals with Crohn's disease includes the use of Tumor necrosis factor-a (TNF-a) inhibitors, such as Infliximab (Remicade^®^). However, infusion of this monoclonal antibody induces remission in only 30-40% of cases. Two polymorphisms have been identified on the TNF-a receptor that are associated with drug response, making pharmacogenomic technologies useful in the prediction of treatment efficacy in Crohn's disease patients [76], and in directing drug development.

The development of new drugs targeting diseases that affect less fortunate populations may not be considered profitable for the pharmaceutical companies of developing countries, many of which are still struggling to move beyond the traditional blockbuster model that focuses on the diseases of the rich. The potential of pharmacogenomic technologies to change the research focus of pharmaceutical companies towards the diseases predominant in the developing world is not obvious in the current context.

### 4) Feasibility and timeliness of implementation

One major limitation of emerging technologies is their successful implementation. To evaluate this factor, the Toronto group proposed that these technologies should have an impact on the health of developing world populations in a reasonable time frame (i.e., 5 to 10 years). The health needs of developing world populations are great, and so technologies should be evaluated (and prioritised) based on their potential for timely implementation.

A number of pharmacogenomic drugs (e.g., Herceptin, Gleevec, Velcade or Erbitux) and drug sensitivity tests (e.g., for abacavir, tamoxifen or warfarin) have been developed. Nonetheless, the relative success of pharmacogenomics technology implementation in the drug development and distribution process is still limited [78]. The low level of development of these technologies is even more evident when attention is focused on the needs of developing world populations. Only one of the 14 drugs approved by the FDA for which pharmacogenomics data are available - i.e., abacavir, a treatment for HIV+ patients - is geared specifically towards meeting the needs of populations in the developing world. There has been little success (or interest?) in gathering relevant pharmacogenomic information on drugs presently in use. Some progress appears to be happening with cancer treatments, with 7 of the 14 drugs for which pharmacogenomic tests are available being geared towards cancer; but the same cannot be said for diseases prevalent in the developing world.

The translation of scientific research into drug discovery and development is a process that can take between 5 to 10 years. Pharmacogenomic technologies are for the most part still in the early stages of development, so it is possible that their successful implementation in drug development is not yet feasible, nor easy to measure accurately. An interim evaluation may be possible, however, by examining the place of pharmacogenomics science in the global health research context. For example, qualitative and quantitative data on the amount and type of research being done using pharmacogenomic technologies (e.g., through an analysis of scientific publications) could provide some indication of whether pharmacogenomics research is actually targeting the needs (e.g., diseases) of developing world populations. The current reality, however, is that pharmacogenomic technologies have not yet met the requirement for successful implementation in a reasonable time frame as proposed by the Toronto group.

### 5) Capacity to reduce the knowledge gap (provide knowledge advancement)

Pharmacogenomic information, especially about population variations in genomic polymorphisms that relate to drug metabolism, may allow for the rapid and efficient screening of a variety of drugs that are in use or in development, in order to identify the best correspondence between treatment, disease and population. The international HapMap consortium sought to develop such information for four populations of African, Asian and European ancestry [79]. More than 4 million single nucleotide polymorphisms (SNPs) were initially identified, providing valuable information for gene expression variation and pharmacogenomic studies [60,80]. However, these SNPs were mostly from people of Caucasian descent, which created a genomics knowledge gap in the translation of this science at a global level [81]. Phase 3 of the project was thus extended to include 11 global populations (International Hap Map Consortium 2010), and 26 million SNPs have now been genotyped, thereby allowing genomic studies in peoples from diverse ethnic backgrounds [81-84].

The information provided remains sparse, with a little over 1000 individuals having had their DNA genotyped during the project. It is thus still very difficult to extrapolate from these small samples to full populations, or to translate the scientific knowledge into clinically applicable information. Other initiatives, such as the 1000 Genome Project (to genotype the full genome of over a 1000 individuals worldwide) can contribute to gathering more of this valuable genomic information [85]. Nonetheless, the high costs and technological requirements associated with these projects suggest that research centres based in developing countries may not easily contribute to nor benefit from the information obtained. Three upper middle-income countries - Brazil, South Africa and Mexico - and two lower middle-income countries - India and Thailand - have developed a research framework that allows the integration of genomic technologies into their national scientific research settings [16,18,86,87]; such infrastructure and human resources are simply not present in low-income countries. This situation introduces a technological gap between scientists from the developed and developing worlds and is likely to reduce the amount of information gathered concerning the populations of these latter countries.

The creation of a knowledge gap in genomics research capacity can translate into asymmetrical pharmacogenomics drug R&D that favours those populations for which more data are available. The geographic locations in which pharmacogenomics research centres operate thus becomes an important factor in defining the populations that will benefit from discoveries in pharmacogenomics. The impact that genomics knowledge can have on public health depends to a large extent on how and why this information influences drug R&D. The use of genomic information on the vulnerability of a population to an HIV treatment, for example, may be very beneficial if it encourages the development of alternate treatments that are safer and more effective for a given population. However, the same information could negatively impact public health if it were used to reduce treatment opportunities, for example, where novel treatments are developed only for populations that show certain genomic characteristics. To cite an old adage, "knowledge is power", and in the case of pharmacogenomics, much will depend on what knowledge is collected and who has the power to put it to use.

### 6) Capacity for indirect benefits

Direct benefits of developing and distributing novel pharmacogenomic drugs that better respond to the needs and realities of developing world populations can be easily identified; amongst these are the recognition of specific health needs, faster and safer access to new drugs [88], increased population health [89], reductions in social and global health inequities known to impair individual health opportunities [90-93], and increased market or work opportunities for secondary actors in the drug distribution context (e.g., grocery stores, market stalls, itinerant hawkers or mobile vendors [94]). All these benefits can positively impact local population health, which would undoubtedly be positive for global public health. However, the possibilities that the costs of healthcare provision may increase due to the introduction of pharmacogenomic technologies in the drug development process could counterbalance the previously mentioned benefits. It thus becomes important to evaluate the indirect benefits that could follow from the implementation of pharmacogenomic technologies in the drug development process, in order to better assess their potential in global health.

Two potential indirect benefits are worth considering: 1) the building of a sense of "worthiness" in vulnerable populations, and 2) economic growth for the developing world. The recognition of the specific health needs of populations of low- and middle-income countries by different health actors through the implementation of innovative pharmacogenomic technologies can support a growing sense of worthiness for these populations. Indeed, vulnerable populations (e.g., elderly populations or minorities) can build a collective sentiment of self-esteem when their needs are recognised and addressed [95,96]. This increased self-esteem at the collective level helps build a sense of worthiness and identity in a population that can have significant social impact, such as a reduction in violence [97]. In the context of low or middle-income countries where conflicts often occur, a reduction of violence can be significantly important to increasing the security of the population and enhancing population health. For example, it has been shown that conflict increases the risk of HIV in vulnerable populations, thereby impairing population health in these nations [98]. Conversely, the isolation and rejection that individuals feel in the HIV epidemic increases the level of violence and the risk of conflicts in high prevalence nations [99]. So innovations that indirectly help building a greater sense of worthiness in vulnerable populations, and thus reduce violence and risk of conflict, can contribute to improving global public health.

Furthermore, the social burden that accompanies poor population health can have a significant negative impact on the economy and development of low- and middle-income nations. Poor health can reduce life opportunities for individuals, thereby perpetuating situations of poverty in populations that are already facing limited resources and are in dire need of basic goods such as food, education, shelter and healthcare services [92,93]. At the population level, the collective reductions in individual opportunities translate into a reduced potential for economic development for national governments, because these reduced opportunities significantly decrease the size of the active/working population in these nations.

For example, diseases such as HIV or malaria in sub-Saharan Africa disproportionately affect young adults, are directly linked to and reinforce poverty, and so reduce a nation's competitiveness at the international level [100]. Of the 13 countries with the lowest life expectancy predictions for 2011, 10 have a per capita GDP under $1000 [21,101,102]. Thus the burden of a weak national economy and poor health outcomes seem to correlate directly. There are numerous ways in which these factors can affect one another, and so improvements in population well-being can thus come from initiatives at either or both levels. The development of drugs that can increase the potential for better health or better response to treatments in the population, and thus contribute indirectly to the stabilization of the human capital of a nation, then become a social necessity. In this view, drugs that may at first appear to constitute a *luxury *(e.g., pharmacogenomic drugs) may actually be a *necessity*.

## Conclusions

The current economic world order is based on principles of globalization that have made it possible for pharmaceutical companies and their shareholders to become extremely prosperous [22]. Pharmaceutical companies engage in drug development in order to both address the health needs of populations, and to contribute to economic well being through wealth creation; and as a result of current drug marketing models (i.e., strong IPR, high priced drugs), the needs of people in developed countries are invariably favoured over those in developing countries. In this context, it remains unclear whether pharmacogenomic technologies can be put at the service of developing world populations to enable more equitable development of drugs that are oriented to meeting the health needs of these neglected populations.

Our application of the University of Toronto Joint Centre for Bioethics criteria (i.e., impact, appropriateness, capacity to address local burdens, implementation in a reasonable time, capacity to reduce the knowledge gap, and capacity for indirect benefits) shows, we argue, that pharmacogenomic technologies have the *potential *to make a significant positive impact on the population health of developing world countries. But in order for this potential to be actualised, the implementation of these technologies in the particular healthcare contexts of developing countries must be carefully scrutinized. There must be further investigation of the questions *how*, *where*, and for *which purpose *are pharmacogenomic technologies being developed? Answering these questions would allow for the identification of activities in pharmacogenomics R&D that show real promise, and so should be favoured (e.g., through technical and financial support) in order to maximise the potential positive impact on the health of low- and middle-income countries. It may also provide crucial information on the responsibilities of the various actors involved in the implementation of pharmacogenomic technologies aimed at meeting the needs of developing world populations.

Pharmacogenomic technologies can contribute to shifting the pharmaceutical industry from its traditional blockbuster model of drug development towards a more personalised and preventive approach. These companies have international activities and so are not limited by national boundaries, policies or realities; as such, they have the potential to make important contributions to improving global population health and ensuring justice in access to needed healthcare services. But given the complexity of genomic information and the still early stage of development of most pharmacogenomic technologies, caution is needed when considering how best to integrate these technologies into the drug development process to ensure that existing social inequities are not exacerbated. In some cases, pharmacogenomic drugs may be a "luxury" given other pressing health needs and the existence of more proven and effective public health interventions. But it is in the development of drugs that are better suited to the specific needs and realities of developing world populations that we see that pharmacogenomic technologies can, in some cases, be considered a *necessary *"*luxury" *in global public health.

## List of abbreviations

ADR: adverse drug effects or reactions; AIDS: acquired immunodeficiency syndrome; ART: anti-retroviral therapies; DNA: deoxyribonucleic acid; FDA: US Food and Drug Administration; HIV: human immunodeficiency virus; IMS Health: Intercontinental Medical Statistics, Health; IMF: International Monetary Fund; IPR: intellectual property rights; MDG: Millennium Development Goals; NGO: non-governmental organisation; PPP: public-private partnership; R&D: research and development; SNPs: single nucleotide polymorphisms; TRIPS: Trade Related Aspects of Intellectual Property Rights; WTO: World Trade Organization.

## Competing interests

The authors declare that they have no competing interests.

## Authors' contributions

CO conceived the study design, analysed the data and wrote the first draft of the manuscript. BWJ helped with the analysis and revision of the manuscript. Both authors read and approved all versions of the text, as well as the final manuscript.
